# An Outbreak of *Clostridium* (*Clostridioides*) *difficile* Infections within an Acute and Long-Term Care Wards Due to Moxifloxacin-Resistant PCR Ribotype 176 Genotyped as PCR Ribotype 027 by a Commercial Assay

**DOI:** 10.3390/jcm9113738

**Published:** 2020-11-20

**Authors:** Elena Novakova, Nina Kotlebova, Anezka Gryndlerova, Martin Novak, Michala Vladarova, Mark Wilcox, Ed Kuijper, Marcela Krutova

**Affiliations:** 1 Department of Microbiology and Immunology, Comenius University Jessenius Faculty of Medicine, 036 01 Martin, Slovakia; elena.novakova@uniba.sk (E.N.); nina.kotlebova@gmail.com (N.K.); martin4novak@gmail.com (M.N.); 2 Department of Medical Microbiology, Charles University, 2nd Faculty of Medicine and Motol University Hospital, 150 06 Prague, Czech Republic; anezkaa.gry@gmail.com; 3 Department of Clinical Microbiology, Clinical Biochemistry Inc., 012 07 Zilina, Slovakia; michala.vladarova@gmail.com; 4 Healthcare Associated Infection Research Group, Leeds Teaching Hospitals NHS Trust & University of Leeds, Leeds LS2 9JT, UK; Mark.Wilcox@nhs.net; 5 Department of Medical Microbiology, Leiden University Medical Centre, 2300 Leiden, The Netherlands; e.j.kuijper@lumc.nl

**Keywords:** ribotyping, Slovakia, MLVA, Thr82Ile, *tcdC*, Δ117, binary toxin

## Abstract

We aimed to characterize *Clostridioides difficile* isolates cultured during a six-month single-center study from stool samples of patients with *C. difficile* infection (CDI) genotyped by the Xpert^®^ *C. difficile*/Epi assay by polymerase chain reaction (PCR) ribotyping, toxin genes’ detection and multi-locus variable number tandem repeats analysis (MLVA). The susceptibility to metronidazole, vancomycin and moxifloxacin was determined by agar dilution. In addition, the presence of Thr82Ile in the GyrA and a single nucleotide deletion at position (Δ117) in the *tcdC* gene were investigated. Between January 1 and June 30, 2016, of 114 CDIs, 75 cases were genotyped as presumptive PCR ribotype (RT) 027 infections using a commercial assay. *C. difficile* isolates cultured from presumptive RT027 stool samples belonged to RT176. These isolates carried genes for toxin A (*tcdA*), B (*tcdB*), binary (*cdtA*/*B*) and had Δ117 in the *tcdC* gene. Using MLVA, the 71/75 isolates clustered into two clonal complexes (CCs). Of these, 39 isolates (54.9%) were from patients hospitalized in acute care and 32 isolates (45.1%) were isolated from patients hospitalized in the long-term care department. All isolates were susceptible to metronidazole and vancomycin, and 105 isolates were resistant to moxifloxacin (92%) carrying Thr83Ile in the GyrA. An outbreak of RT176 CDIs, suspected as RT027, was recognized in a Slovakian hospital. In order to monitor the emergence and spread of RT027-variants, the identification of a presumptive RT027 CDI should be confirmed at a strain level by PCR ribotyping.

## 1. Introduction

*Clostridium difficile*, recently reclassified as *Clostridioides difficile*, belongs to the most important healthcare-associated pathogens [1,2]. The major virulence factors in *C. difficile* are toxins TcdA and TcdB; some strains can produce a third toxin, *C. difficile* transferase (CDT) which is named binary toxin [1,3]. Toxin synthesis is controlled by a regulatory network and is dependent on the availability of nutrients [4]. The *tcdC* gene, located in the pathogenicity locus (PaLoc), encodes one of the proteins involved in transcriptional regulation [4,5].

*C. difficile* infection CDI) epidemiology has changed in the last two decades due to the spread of epidemic ribotypes (RTs), e.g., RT027 [6,7]. RT027 carry toxin genes for the expression of all three known toxins (A, B and binary), [7]. Moreover, in RT027, a single nucleotide deletion at position 117 causing a frameshift that introduces a stop codon in the *tcdC* has been detected [7,8]. The subsequent truncation of TcdC has been suggested as a reason for increased toxin production [8]. The epidemiological importance of RT027 led to the development of several commercial molecular assays for the differentiation of *C. difficile* RT027 from other ribotypes. The assays are based on the presence of toxin genes (toxin B, binary toxin) and the specific deletion at position 117 in the *tcdC* gene [9].

In response to the increasing CDI incidence, the European Centre for Disease Prevention and Control (ECDC) started to coordinate hospital-based CDI surveillance in European Union/European Economic Area (EU/EEA) countries from January 2016 and it includes acute and long-term care. The enhanced CDI surveillance option requests microbiological data, i.e., the ribotype, the presence of toxins/toxin genes and antimicrobial susceptibility testing [6].

In order to map the CDI epidemiology in a Slovak hospital, a rapid molecular test for a presumptive RT027 identification from stool samples was implemented. Due to the high proportion of presumptive RT027 stool samples identified, we aimed to characterize *C. difficile* isolates to confirm these results at a *C. difficile* strain level.

## 2. Material and Methods

### 2.1. Diagnostics of CDI

The six-month study was carried out from 1 January to 30 June 2016 in a tertiary-care center in the northern part of Slovakia. The hospital had 726 beds and 12,575 admissions during the study period.

Unformed stool samples from patients with a suspected CDI were tested for the presence of toxins A/B (CerTest *Clostridium difficile* GDH + Toxin A + B one-step combo card test, Zaragoza Spain). The toxin A/B-positive stool samples were investigated using the Xpert^®^ *C. difficile*/Epi assay (Cepheid, Sunnyvale, CA, USA) that detects the presence of *tcdB* (toxin B) and *cdtB* (binary toxin) genes and the deletion at position 117 in the *tcdC* gene (glycosylating toxin anti-sigma factor). All toxin A/B-positive stool samples were simultaneously cultured anaerobically on selective media for *C. difficile* (Brazier, Hampshire, Oxoid, UK).

### 2.2. Molecular Typing of Isolates

*C. difficile* isolates were characterized further by polymerase chain reaction (PCR) capillary-based electrophoresis ribotyping [10], detection of toxin genes (*tcdA*—toxin A, *tcdB*—toxin B, *cdtA* and *cdtB*—binary toxin) [11] and sequencing of the *tcdC* gene fragment [12] to confirm the presence of a single nucleotide deletion at position 117 (∆117). To reveal the genetic relatedness of isolates with a predominating RT, a multi-locus variable number tandem repeats analysis (MLVA) of seven previously published regions with short tandem repeats was applied [13]. In selected isolates, multi-locus sequence typing using seven housekeeping genes (MLST) was also performed [14].

### 2.3. Antimicrobial Susceptibility Testing

Antimicrobial susceptibility to metronidazole, vancomycin and moxifloxacin was determined by agar dilution on Wilkins Chalgren agar (Hampshire, Oxoid, UK). The clinical *C. difficile* isolate with defined minimum inhibitory concentration (MIC) values by the E-test was used as a control. The MIC breakpoints for metronidazole (2 mg/L), vancomycin (2 mg/L) and moxifloxacin (4 mg/L) as recommended by the European Committee on Antimicrobial Susceptibility Testing (EUCAST) were applied. In addition, the presence of an amino acid substitution Thr82Ile in the GyrA was investigated as a related molecular resistance mechanism [15].

## 3. Results

### 3.1. Presumptive Ribotype 027 CDI Outbreak Was Identified from Stool Samples

**Figure 1:**
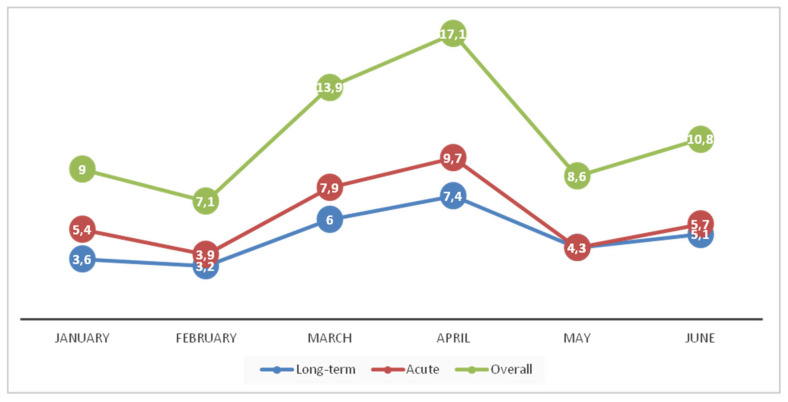
*Clostridioides* (*Clostridium*) *difficile* infection incidence during the study period.

During the six-month period (1 January to 30 June 2016), 114 stool samples proved positive for the presence of toxins A/B. The mean six-month CDI incidence was 11.1 cases/10,000 patient-days, ranging from 7.1 to 17.1 cases/10,000 patient-days (Figure 1). Sixty-seven samples were from females (58.8%) and the median age of the patients was 77.5 years. Based on CDI origin [6], 88 cases were healthcare-associated (77.2%), one case was community-associated and 25 patients had a recurrent CDI (21.9%). Ten patients died (8.8%).

Molecular genotyping of toxin A/B-positive stool samples revealed that 114 samples were positive for the presence of the *tcdB* gene, and 75 (65.8%) stool samples were positive for all three commercial assay target sites in the *C. difficile* genome: *tcdB*, *cdtB* and ∆117 in the *tcdC* gene, and were assigned as presumptive *C. difficile* RT027 samples.

### 3.2. Suspected C. difficile RT027 in Stools Were Identified as C. difficile RT176 at a Strain Level

**Figure 2:**
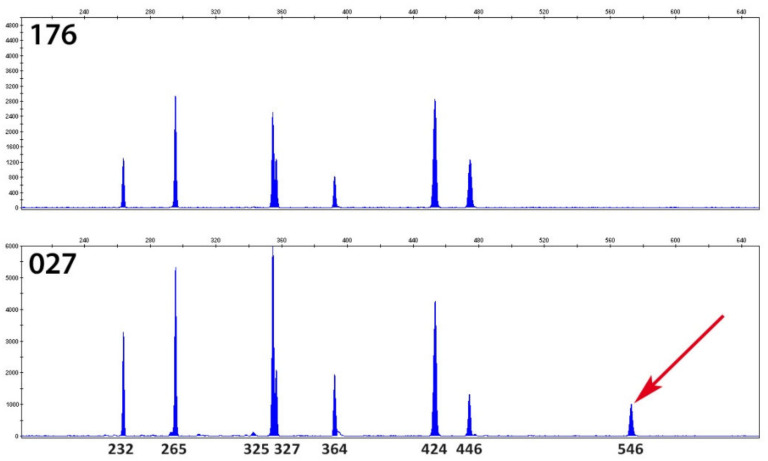
The capillary electrophoresis PCR ribotyping patterns of ribotype 027 and 176. Fragment analysis was performed on an ABI 3130xl DNA analyzer (Applied Biosystems) with the LIZ 1200 size standard (Applied Biosystems). The electrophoreograms were visualized in Gene Mapper v5.0 (Applied Biosystems). The numbers indicate the length of individual fragments in bases.

**Figure 3:**
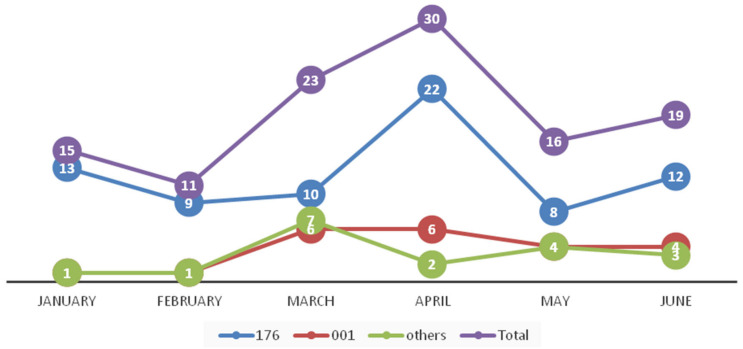
The distribution of ribotypes during the study.

PCR ribotyping of *C. difficile* isolates cultured from presumptive PCR ribotype 027 stool samples (*n* = 75) revealed that the *C. difficile* isolates belonged to RT176 (Figure 2, the differences between RTs 027 and 176 ribotyping patterns). Other RTs identified from non-RT027 stool samples were: 001 (*n* = 22, 19.3%), 017 (*n* = 8, 7.0%), 002 and 014 (*n* = 2, 1.8%, each), 011, 012, 031, 081 and unknown (*n* = 1, 0.9%, each). The distribution of RTs during the study period is shown in Figure 3.

Toxin genes’ multiplex PCR showed that 113 (99.1%) isolates were positive for the presence of the genes for toxin A and B (*tcdA* and *tcdB*). Of them, 75 (65.8%) isolates (RT176) also carried the genes for the binary toxins (*cdtA* and *ctdB*) and had the ∆117 in the *tcdC* gene. The remaining one isolate revealed the absence of toxin genes and the *tcdC* gene (RT031).

### 3.3. Moxifloxacin Resistance Due to Thr82Ile Was Identified in Majority of Isolates

All isolates were susceptible to metronidazole and vancomycin. One-hundred and five isolates were resistant to moxifloxacin (92.1%) with the presence of Thr82Ile substitution in the GyrA: RTs 176 (75/75), 001 (21/22), 017 (8/8), 002 (1/2).

### 3.4. Clonal Relatedness of RT176 Isolates Were Identified from Samples within Acute and Long-Term Care Wards

**Figure 4:**
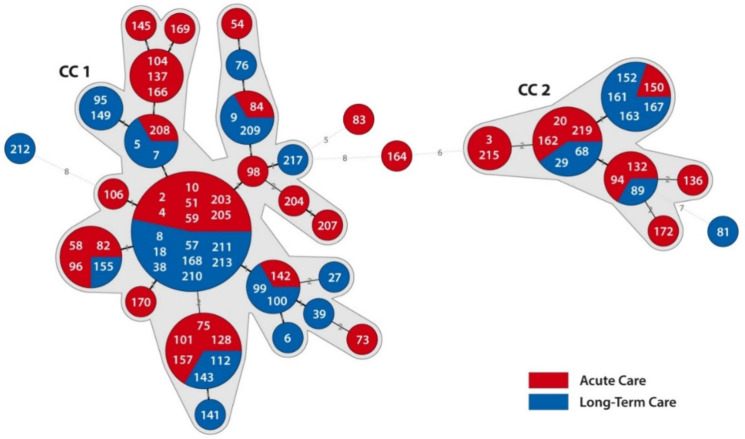
Minimum spanning tree (MST) of *Clostridioides* (*Clostridium*) *difficile* isolates of ribotypes 176. A MST was created by Bionumerics v5.0 (Applied Math) using the Manhattan coefficient. The numbers in circles represent the DNA number of *C. difficile* isolates. The numbers on the lines represent the sum of tandem repeat differences (STRD) between isolates. If more than one number is present in one circle, it represents isolates with STRD = 0. A clonal complex (CC) was defined as STRD ≤ 2. The red color indicates *C. difficile* isolates derived from patients hospitalized in acute care wards and the blue color represents *C. difficile* isolates derived from patients hospitalized in a long-term care ward.

MLVA of *C. difficile* RT176 isolates revealed two clonal complexes (CCs). CC1 comprised 54 isolates and CC2 comprised 17 RT176 isolates. Only 4 isolates showed no clonal relatedness but their Summed tandem-repeat differences (STRD) did not exceeded 10. Of clonal-related RT176 isolates, 39 (54.9%) were derived from patients hospitalized in acute care and 32 (45.1%) were derived from patients hospitalized in a long-term care department (Figure 4). MLST of 16 selected isolates within and out of CCs showed that all isolates belonged to ST1 and Clade 2.

### 3.5. RT176 Infection Is Not Associated with Fatal Outcome or Recurrence of CDI

A fisher exact test showed no association between RT176 CDIs and fatal outcome of patients (6/69 vs. 4/35, *p* = 0.7335) or recurrence of CDI (16/59 vs. 9/30, *p* = 0.8162).

## 4. Discussion

This six-month single-center study showed the mean CDI incidence of 11.1 cases/10,000 patient-days that was five times higher than hospital laboratory-based CDI incidence in 2015 and four times higher than CDI incidence that was reported for 36 Slovakian hospitals between October and December 2016 [16], suggesting likely healthcare facility transmission of *C. difficile*, especially noting the high prevalence of presumptive ribotype 027 stool samples (*n* = 75, 65.8%).

Surprisingly, the characterization of *C. difficile* isolates by ribotyping, as recommended by the ECDC [6], identified that *C. difficile* isolates cultured from the presumptive ribotype 027 CDIs belong to ribotype 176 (Figure 2 shows the difference in ribotyping profiles of RTs 027 and 176). Using MLVA for the differentiation of highly related and non-related *C. difficile* isolates of the same ribotype [17], the clonal relatedness of *C. difficile* RT176 was detected between isolates from cases in acute care and long-term care wards.

In Slovakia, long-term care facilities serve patients who need rehabilitation or palliative care. Admission to an acute care department usually precedes admission to a long-term care facility, but due to the clinical deterioration of patients, a re-transfer to an acute care department can be requested. Our data suggest that the spread of *C. difficile* RT176 was facilitated via the transfer of patients between hospital acute care and long-term care wards. This is supported by data on previous hospitalization of patients in this study. Of 75 patients infected by *C. difficile* RT176, 31 cases had hospitalization in acute care wards in the four weeks prior to their stay in a long-term care ward (and vice versa in one patient).

**Figure 5:**
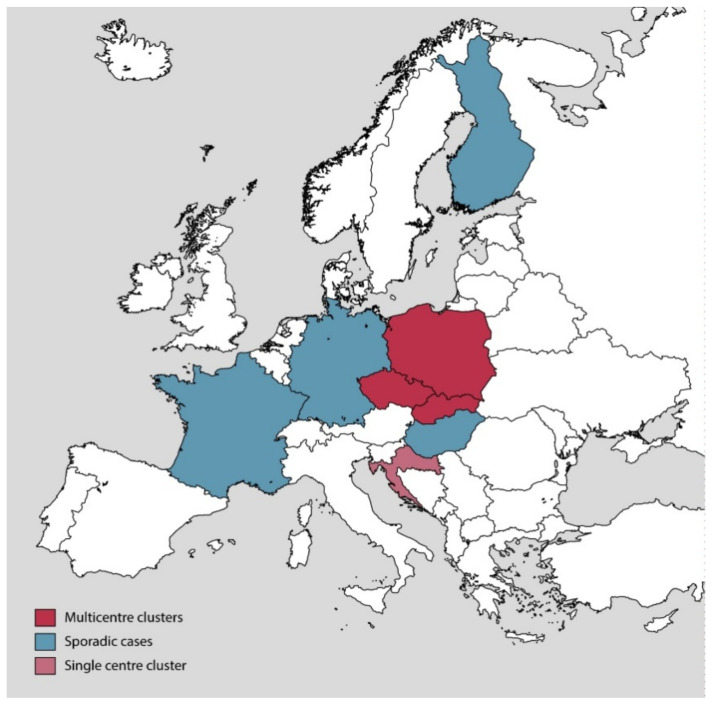
European countries where the PCR ribotype 176 was reported. Sources of information include reports from: Czech Republic [19], Croatia [21], Hungary [23], Germany [20], Finland [22], France [24], Poland [18] and Slovakia [16].

Our study identified 75 cases of RT176 CDI between January and July 2016. Later in the year, between October and December 2016, another 23 cases from five Slovakian hospitals were reported during the participation of Slovakia in the European standardized CDI surveillance [16], which indicates the possible dissemination of RT176 across healthcare facilities in the country. In addition to Slovakia, the epidemiologically significant occurrence of RT176 has been reported in its neighboring countries, Poland and the Czech Republic [18,19], as well as other countries in Europe: Germany, Croatia, Finland, Hungary and France [20,21,22,23,24] (Figure 5).

The genetic similarity between RTs 176 and 027 in chromosome loci used by commercial assays for differentiating of RT027 has already been reported in Czech and Finnish *C. difficile* isolates [22,25]. Recently, the misidentification of RT027 by a commercial assay targeting *tcdB*, *cdtB* and Δ117 in the *tcdC* gene was reported in RT244 CDIs from Australia [26] and recently in RT591 CDIs in the USA [27]; however, those RTs belonged to a different sequence type (41) but in the same clade 2. Although there is a close genetic similarity between RT176 and the “hypervirulent” 027 ribotype [28], a significant association between RT176 CDIs and fatal outcome of patients or recurrence of CDI was not confirmed. However, the high transmissibility/spread potential of RT176 in this outbreak was evident given its high prevalence (65.8%).

In addition to the genetic relatedness of RT176 to epidemic RT027, antimicrobial resistance undoubtedly plays an important role in the spread of *C. difficile* strains [29]. In our RT176 isolates, the resistance to moxifloxacin was due to the well-described alteration at the Thr82Ile in the GyrA, fluoroquinolones target site [7]. In contrast, isolates of RTs 244 and 591 were moxifloxacin-susceptible, and it is notable that these have been reported less frequently than RT176 [21,22,23].

## 5. Conclusions

An outbreak of moxifloxacin-resistant RT176 CDIs was recognized in a Slovakian hospital. Due to a genetic similarity to RT027, the identification of a presumptive ribotype 027 *C. difficile* based on the presence of toxin genes (toxin B, binary toxin) and the specific deletion at position 117 in the *tcdC* gene has to be confirmed on a strain level by PCR ribotyping in order to monitor the emergence and spread of RT027 variants.
